# Comparing implantable epimysial and intramuscular electrodes for prosthetic control

**DOI:** 10.3389/fnins.2025.1568212

**Published:** 2025-06-16

**Authors:** Eric J. Earley, Morten B. Kristoffersen, Max Ortiz-Catalan

**Affiliations:** ^1^Department of Electrical Engineering, Chalmers University of Technology, Gothenburg, Sweden; ^2^Center for Bionics and Pain Research, Mölndal, Sweden; ^3^Department of Orthopedics, University of Colorado Anschutz Medical Campus, Aurora, CO, United States; ^4^Bone-Anchored Limb Research Group, University of Colorado Anschutz Medical Campus, Aurora, CO, United States; ^5^Department of Orthopaedics, Institute of Clinical Sciences, Sahlgrenska Academy, University of Gothenburg, Gothenburg, Sweden; ^6^Prometei Pain Rehabilitation Center, Vinnytsia, Ukraine; ^7^Center for Complex Endoprosthetics, Osseointegration, and Bionics, Kyiv, Ukraine

**Keywords:** implantable electrode, myoelectric control, intramuscular, epimysial, prosthetics, neural interface

## Abstract

**Introduction:**

Implantable electrodes are the subject of increasing interest due to the possibilities they present for the control of assistive devices such as prosthetic limbs, however evidence as to whether epimysial or intramuscular electrodes result in better performance is lacking.

**Methods:**

In this retrospective study, we analyzed data collected from six users of a neuromusculoskeletal arm prosthesis who had been implanted with epimysial or intramuscular electrodes, two of whom were implanted with both electrode types, and compared electrical impedance and electromyographic feature space characteristics – all important qualities for the control of prosthetic arms and hands.

**Results:**

Our results showed significantly greater cross-channel impedances for intramuscular electrodes suggesting improved isolation and reduced cross-talk, however this did not translate to significantly improved signal-to-noise ratio, separability, or repeatability. Sequential feedforward selection analysis may suggest that epimysial electrodes contribute greater signal separability when recording from larger muscles used for gross hand movements, whereas intramuscular electrodes contribute greater signal separability when recording from smaller muscles used for grasp prehension and finger movements, but additional study is required to confirm these findings.

**Discussion:**

Our results provide preliminary understanding as to which electrodes should be used for which patients, which may help to guide clinical practice for future implementation of cutting-edge bionic arms.

## 1 Introduction

Prosthetic arms and hands are becoming rapidly more advanced and increasingly more available to those with upper-limb amputations. This increased complexity has the potential to confer great benefits to these individuals, increasing personal functionality and improving their quality of life. However, the control of such devices is still limited by a number of factors, chief among them a lack of high-quality neural signals to drive the prosthesis (Al-ajam et al., 2022; Zbinden et al., 2023). Most prosthetic hands are controlled using surface electromyography (sEMG) placed over agonist-antagonist muscle pairs, and are capable of little more than opening and closing; for multifunction bionic hands capable of separate finger actuations, selecting different grasping patterns is typically done using set patterns of co-contraction (Jaramillo-Yánez et al., 2020), motion control, a mobile app, or near-field communication (NFC) functionalities (Igual et al., 2019; Roche et al., 2014). Moreover, sEMG is highly susceptible to motion artifacts and electromagnetic interference that reduce the operational range of myoelectric signals as floor noise thresholds must be raised to decrease the instances of undesired actuations. Implanted electromyography (iEMG) electrodes have been shown to provide lager operational ranges and improved reliability (Ortiz-Catalan et al., 2019; Ortiz-Catalan et al., 2020), while also providing access to deeper muscles effectively increasing the neural information available for prosthetic control as compared with sEMG.

The benefits of implantable electrodes are manyfold. Aside from providing larger operational ranges and reduction of motion artifacts, implantable electrodes can be used for targeted recording of specific muscles. Although surface electrodes are capable of targeting large muscle pairs (e.g., biceps and triceps), their ability to capture specific myoelectric activity is significantly reduced when multiple smaller muscles are present, or when the muscles of interest are obstructed superficially by other muscles (e.g., wrist flexor and extensor muscles lie superficial to the finger flexors and extensors). Activation of superficial muscles can impair the ability for pattern recognition decoders to reliably predict the intended motions of underlying muscles (Earley and Hargrove, 2016; Fougner et al., 2011; Khushaba et al., 2014; Pan et al., 2014), necessitating longer additional training data sessions or sensor fusion approaches to achieve similar performance (Adewuyi et al., 2017; Earley et al., 2016). Implantable electrodes can bypass this limitation by directly measuring signals from deep muscles. This benefit also extends to neuromuscular constructs resulting from reconstructive surgical procedures such as targeted muscle reinnervation (TMR) (Kuiken, 2003) or regenerative peripheral nerve interfaces (RPNIs) (Al-ajam et al., 2022), which create additional myoelectric motor signals (Kuiken et al., 2009; Miller et al., 2008; Vu et al., 2023).

In addition to muscle targeting, implantable electrodes circumvent many of the limitations of surface electrodes. For example, the shifting of electrodes on the surface of the skin has been known to deteriorate prosthesis control, as does electrode liftoff (Simon et al., 2023). If electrodes are embedded in a prosthetic socket, the socket may be uncomfortable due to chafing and sweating. Even if a socket is not used, sEMG signals can be affected by dry or hairy skin, or by the ambient temperature (for example, the frigid north of Swedish Lapland). Implantable electrodes are not affected by these conditions, making them a robust option for use with bionic hands. Previous studies have characterized the benefits of implantable electrodes over surface electrodes (Farrell and Weir, 2008; Hargrove et al., 2007; Mastinu et al., 2019; Perry et al., 1981; Smith and Hargrove, 2013), however these studies do not consider the different types of implantable electrodes.

There are two main types of implantable electrodes: epimysial electrodes and intramuscular electrodes. Epimysial electrodes are sutured onto the epimysium of the target muscle and do not penetrate the muscle tissue. Intramuscular electrodes, as the name suggests, are inserted into the muscle tissue itself, directly recording from within the muscle. Due to differences in geometry and size (epimysial electrodes tend to be larger than intramuscular electrodes), the decision as to which electrode to use is sometimes clear – for example, epimysial electrodes compromise RPNI constructs as their relatively large size obstructs blood supply. However, in cases where either electrode type would be viable, there is no clear consensus as to which electrode should generally be used for the controlling assistive devices such as bionic hands.

The purpose of this study is to conduct a retrospective analysis of data collected from individuals with implanted epimysial and intramuscular electrodes to determine if either electrode type can be considered more generally suitable for the control of a prosthetic hand. Electrical impedance and EMG feature space characteristics were calculated to present evidence of superiority for prosthesis use. Our results suggest that intramuscular electrodes may generally be better at isolating the signal from the target muscle and rejecting non-target signals, but that epimysial electrodes enable better prosthesis control when recording from larger and more isolated muscles. Although only two of the included individuals have been implanted with both electrode types, with all others only using one type or the other, the analysis of *in vivo* data from long-term users presented herein offers promising preliminary results which may guide clinical practice and future investigations.

## 2 Materials and methods

### 2.1 Participants

The data from six individuals who had been implanted with epimysial or intramuscular electrodes as part of receiving a neuromusculoskeletal prosthesis (Integrum AB, Mölndal, Sweden) were collected and analyzed in this study. In addition to the implanted electrodes, the neuromusculoskeletal prosthesis also comprise titanium intermedullary screw-fit implants affixed to the bone(s) of the residual limb for prosthesis suspension, and spiral cuff electrodes affixed to the remaining nerves for sensory feedback via electrical stimulation (Earley et al., 2024; Ortiz-Catalan et al., 2020; Ortiz-Catalan et al., 2023; Zbinden et al., 2023). Relevant details of the six individuals are summarized in Table 1.

**TABLE 1:** Summary of participants and implanted electrodes.

Participant*	Sex	Years since amputation	Years since electrode implantation	Epimysial electrodes	Epimysial configuration	Intramuscular electrodes	Notes**
TH1	M	7	1	8	Bipolar	–	
TH2	M	5	2	4	Monopolar	8	3 TMR (2 Epimysial, 1 Intramuscular); 5 RPNI
TH3	M	21	1	8	Bipolar	–	4 TMR
TH4	M	16	6	8	4 Monopolar, 4 Bipolar	–	1 TMR
TR1	F	19	3	4	Monopolar	8	4 RPNI
TR2	M	25	2	–	–	12	

*TH, trans-humeral amputation; TR, trans-radial amputation.

**TMR, targeted muscle reinnervation; RPNI, regenerative peripheral nerve interface.

This study was conducted in accordance with the terms of the Declaration of Helsinki. The study was approved by the Swedish ethical review board (Dnr: 2020-04600); all individuals provided informed consent prior to participation in the research study to receive the neuromusculoskeletal prosthesis.

### 2.2 Electrodes

**FIGURE 1:**
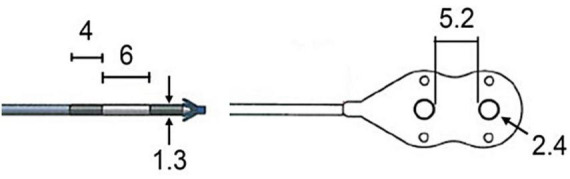
Neuromusculoskeletal prosthesis users had intramuscular **(left)** and epimysial **(right)** electrodes implanted in their residual limbs for the purposes of prosthesis control All dimensions are in millimeters.

Figure 1 shows photos of the two electrode types investigated in this study. Five individuals were implanted with epimysial electrodes (Figure 1, right). Each epimysial electrode contact is composed of 90% platinum and 10% iridium (PT90/IR10) with the electrode body made of silicone. Epimysial electrodes have a diameter of 2.4 mm and a surface area of 4.5 mm^2^ with an inter-electrode distance of 5.2 mm.

Three individuals were implanted with intramuscular electrodes (Figure 1, left) composed of stainless steel 316 LVM wire with silicone tubing for insulation. Intramuscular electrodes have a diameter of 1.3 mm and a surface area of 17.7 mm^2^ with an electrode length of 4 mm and an inter-electrode distance of 6 mm.

Two individuals were implanted with both epimysial and intramuscular electrodes. Of the three individuals with only epimysial electrodes, two had their electrodes in a bipolar configuration, and one had their electrodes in both bipolar and monopolar configurations; the monopolar epimysial electrodes had the same layout and dimensions as the bipolar electrode shown in Figure 1, but without the second electrode surface. Electrode implantation location was based primarily on functional expectations for prosthetic control, balancing factors including recording from muscles most used in distal movements, muscle size, and aiming to target as many independent muscles as possible.

We employed three measures to quantify the performance of the two electrode types. Impedance is a measure of how much an electrode is able to resist the flow of electrical current - a higher impedance between two electrodes indicates that less current will flow between the electrodes. Here, we differentiate between direct impedance (measured between an electrode and its electrical ground) and cross-channel impedance (measured between two electrodes) (Earley et al., 2022a). High direct impedance can be detrimental for recording biosignals, but higher cross-channel impedance can be beneficial for avoiding cross-talk between electrodes. Another method by which signal quality can be compared between electrode types is by calculating the signal-to-noise ratio (SNR). A higher SNR indicates that an electrode is better able to distinguish the contraction of its target muscle during a specific movement, compared to the ambient signals during rest.

For pattern recognition based control of a prosthetic limb, the EMG feature space characteristics are important measures. EMG features should be repeatable for each movement class, while being distinct between movements classes. Separability is a measure of how dissimilar the measured iEMG signals are when performing different movements – for example, opening and closing the hand. Greater separability is indicative of an improved ability for pattern recognition algorithms to differentiate between movement classes while controlling the prosthetic hand. Likewise, repeatability is a measure of how similar the measured iEMG signals are when performing the same movements multiple times. Greater repeatability is indicative of improved prosthesis control and a reduced need to retrain the pattern recognition algorithm (Kristoffersen et al., 2019).

#### 2.2.1 Impedance

Electrode impedance was measured to monitor their connectivity and to identify and diagnose issues with signal quality. To do so, a sinusoidal current of known amplitude and frequency (38.4 nA at 1 kHz, 19.2 nA at 500 Hz) was applied between the electrode and the reference, and the resulting voltages were measured. The impedance (at the given frequency) can then be calculated according to Ohm’s law by dividing the voltage amplitude by the current amplitude.

By taking voltage measurements across every pair of connectors (electrodes plus reference), it is possible to determine two types of impedance: direct impedance and cross-channel impedance (Earley et al., 2022a). Direct impedance is the impedance between an electrode and the reference, and cross-channel impedance is the impedance between two electrodes. For the purposes of measuring iEMG for prosthesis control, all electrodes are electrically isolated from one another. A large ratio between cross-channel impedance and direct impedance would indicate precisely this, while a small ratio would indicate that there is substantial cross-talk between electrodes – in other words, that they are measuring the same source and are therefore not independent.

Due to impedance measurement errors, data for TH2 and TH3 were corrupted and are therefore not included in this analysis.

#### 2.2.2 Signal-to-Noise ratio

Recording sessions from each participant were identified where they performed either gross hand movements (open/close hand, flex/extend wrist, pronate/supinate wrist and (for participants with transhumeral amputation) flex/extend elbow) or finger movements (flexion/extension of each finger). EMG was sampled at 500 Hz (TH2, TR1, TR2), 1 kHz (TH1, TH3), or 2 kHz (TH4). Steady-state EMG signals were isolated during movements, and the SNR (in decibels) was calculated as:


(1) $$S\,N\,R_{d\,B}=10*\log_{10}\frac{E\,M\,G_{R\,M\,S,m\,o\,v\,e\,m\,e\,n\,t}^{2}}{E\,M\,G_{R\,M\,S,r\,e\,s\,t}^{2}}$$

where EMG_*RMS, movement*_ is the root-mean-square (RMS) of the EMG signal amplitude during a movement and EMG_*RMS, movement*_ is the RMS of the EMG signal amplitude at rest. For each electrode, SNR was calculated across all viable movements; the maximum SNR for each electrode was then computed for both gross movements and finger movements.

#### 2.2.3 Separability and repeatability

Prior to calculating the separability and repeatability, the features must be calculated to define the feature space. The EMG was segmented in 200 ms time windows overlapping with a 50 ms increment. The mean absolute value, slope sign changes, zero crossings, and wavelength were calculated for each time window. The feature space was calculated per participant.

Separability was calculated using Inter-class Distance Nearest Neighbor (IDNN) (Kristoffersen et al., 2019). IDNN is defined as:


(2) $$I\,D\,N\,N_{i}=\min_{i=1,\cdots \,j-1,j+1,\cdots \,m}\frac{d\,i\,s\,t_{j}^{i}*d\,i\,s\,t_{i}^{j}}{d\,i\,s\,t_{j}^{i}+d\,i\,s\,t_{i}^{j}}$$

where *m* is the number of movements, and $d\,i\,s\,t_{j}^{i}$ and $d\,i\,s\,t_{i}^{j}$ are half the Mahalanobis feature space distance between movements *i* and *j*, and *j* and *i*:


(3) d⁢i⁢s⁢tji=12⁢(μT⁢i-μT⁢j)T*ST⁢i-1*(μT⁢i-μT⁢j)

(4) d⁢i⁢s⁢tij=12⁢(μT⁢j-μT⁢i)T*ST⁢j-1*(μT⁢j-μT⁢i)

Repeatability was calculated using Within-Class Distance (WD) (Kristoffersen et al., 2019). WD is defined as:


(5) $$W\,D_{j}=\sum_{k\neq r}^{m}\frac{d\,i\,s\,t_{k\,j}^{r\,j}*d\,i\,s\,t_{r\,j}^{k\,j}}{d\,i\,s\,t_{k\,j}^{r\,j}+d\,i\,s\,t_{r\,j}^{k\,j}}$$

where *r* and *k* are different repetitions of movement *j*. The IDNN and WD are calculated for TH2 and TR1 as they are the only participants in our cohort who have both electrode types on the same muscles. To assess the contribution of each electrode type on separability and repeatability, the IDNN and WD are calculated for each movement with all electrodes except either the intramuscular or epimysial electrode of interest. In this manner, the contribution of either electrode type on the separability can be assessed. Furthermore, sequential forward selection is used to rank all electrodes in terms of their contribution to the separability. To do so, we first identified the single electrode with the greatest separability; additional electrodes are then added, one at a time, by selecting the subsequent electrode which yields the largest improvement in separability.

### 2.3 Statistical analysis

Analyses presented in this study should be considered exploratory due to low sample numbers, however statistical tests are nonetheless used to aid in the interpretation of our results. Wilcoxon rank sum tests are used for paired comparisons of impedances, SNR, and separability and repeatability between epimysial and intramuscular electrodes. Holm-Bonferroni corrections were made to account for multiple comparisons.

## 3 Results

### 3.1 Impedance

**FIGURE 2:**
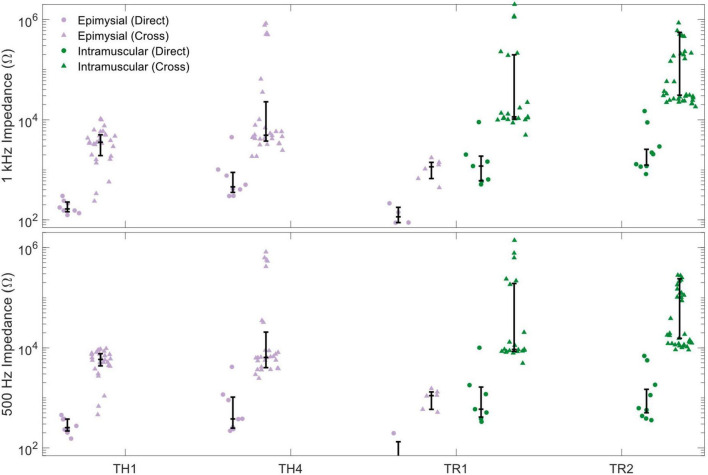
Both direct impedances (circles) and cross-channel impedances (triangles) are higher for intramuscular electrodes (green) than for epimysial electrodes (violet). Impedances are measured both at 1 kHz **(top)** and at 500 Hz **(bottom)**.

Calculated impedances are shown in Figure 2. For impedance measured at 1 kHz (Figure 2), direct impedances were higher for intramuscular electrodes (median [quartiles]: 2.03 kΩ [1.17 kΩ, 8.99 kΩ]) than for epimysial electrodes (227 Ω [148 Ω, 408 Ω], *p* < 0.001); cross-channel impedances were also higher for intramuscular electrodes (212.6 kΩ [23.8 kΩ, ∞Ω]) than for epimysial electrodes (4.17 kΩ [1.99 kΩ, 5.84 kΩ], *p* < 0.001), and were generally at least two orders of magnitude higher than the direct impedances.

The same trends were observed for impedances measured at 500 Hz (Figure 2). Direct impedances were higher for intramuscular electrodes (1.13 kΩ [453 Ω, 6.55 kΩ]) than for epimysial electrodes (248 Ω [199 Ω, 381 Ω], *p* < 0.001); cross-channel impedances were also higher for intramuscular electrodes (148.1 kΩ [12.1 kΩ, ∞Ω]) than for epimysial electrodes (5.84 kΩ [3.67 kΩ, 8.06 Ω], *p* < 0.001).

**FIGURE 3:**
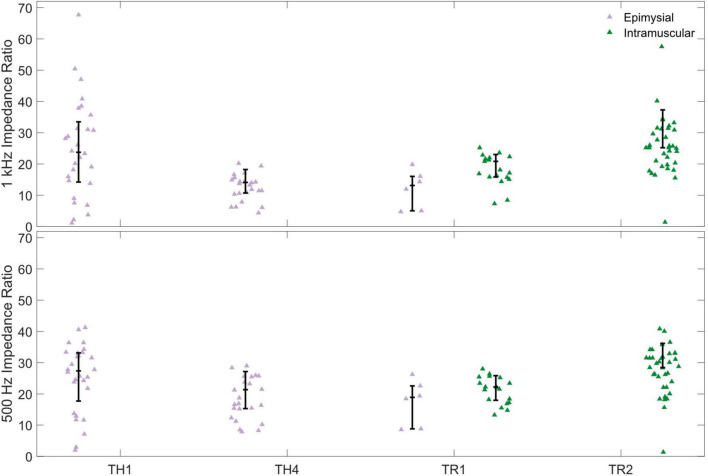
The ratio between cross-channel impedance and direct impedance is higher for intramuscular electrodes (green) than for epimysial electrodes (violet). Impedances ratios are calculated both at 1 kHz **(top)** and at 500 Hz **(bottom)**.

To better understand how changes in both direct and cross-channel impedance may affect prosthesis control, we calculated the ratio between cross-channel and direct impedance. These ratios are shown in Figure 3. Similar to analysis of the calculated impedances, impedance ratios at 1 kHz were higher for intramuscular electrodes (30.9 [21.1, ∞]) than for epimysial electrodes (15.8 [10.8, 28.8], *p* < 0.001); the same trend was seen at 500 Hz (31.6 (23.6, ∞] vs. 24.1 [15.2, 31.6], *p* < 0.001). Overall, these results suggest that while overall impedances are higher for intramuscular electrodes than for epimysial electrodes, the higher impedance ratio for intramuscular electrodes may be better able to electrically isolate signals between electrodes, which could improve the ability to isolate myoelectric source signals.

### 3.2 Signal-to-noise ratio

**FIGURE 4:**
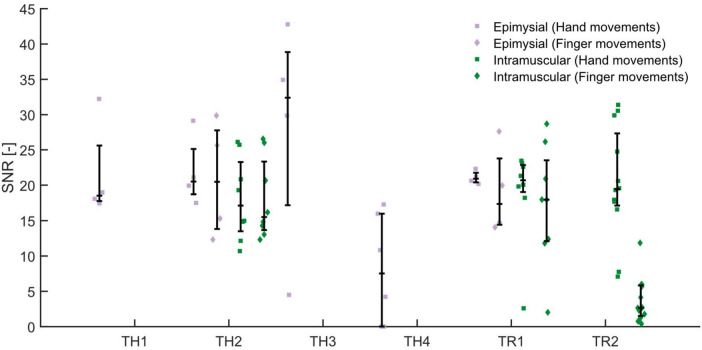
The signal-to-noise ratio (SNR) for a given electrode was calculated as the ratio between the magnitude of the EMG signal during either gross hand (square) or individual finger movements (diamond), and the magnitude of EMG at rest; the movement with the largest SNR is shown in the figure and used for statistical analyses. We found no significant differences in SNR between epimysial (violet) or intramuscular (green) electrodes.

The signal-to-noise ratios calculated during hand and finger movements are shown in Figure 4. For SNR calculated during individual finger movements, we observed a trend toward higher SNR for epimysial electrodes (17.63 [14.43, 26.62]) than for intramuscular electrodes (12.09 [2.67, 17.97], *p* = 0.057). We found no significant differences between epimysial (19.45 [15.99, 22.31]) and intramuscular electrodes (19.68 [15.79, 23.28], *p* > 0.999) when performing gross hand movements. We believe that the trend seen for individual finger movements was largely influenced by patient TR2, who only had intramuscular electrodes and who frequently experienced difficulty performing finger movements. Looking at data from only patients TH2 and TR1, who had been implanted with both electrode types, reveals only negligible differences in SNR during either hand movements or finger movements.

### 3.3 Separability and repeatability

**FIGURE 5:**
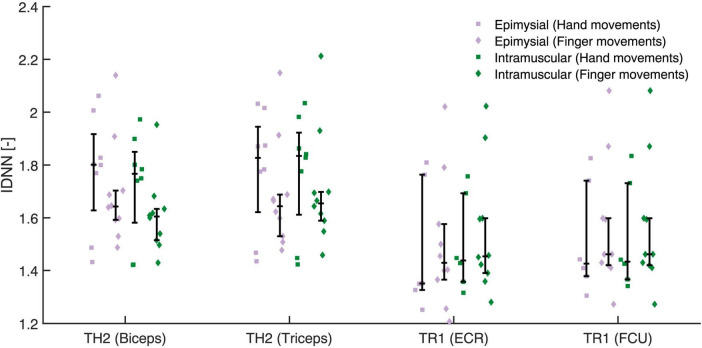
The Inter-class Distance Nearest Neighbour (IDNN) was calculated for either gross hand (square) or individual finger movements (diamond) using all electrodes except either the epimysial (violet) or intramuscular (green) electrode of the targeted muscle (shown in parenthesis next to the participant ID).

Separability calculated with IDNN for hand and finger movements is shown in Figure 5. For hand movements we found no difference between epimysial (1.76 [1.42, 1.82]) and intramuscular electrodes (1.73 [1.42, 1.83], *p* = 0.98). Similarly, for finger movements we found no difference between epimysial (1.59 [1.44, 1.68]) and intramuscular electrodes (1.59 [1.45, 1.67], *p* = 0.837).

**FIGURE 6:**
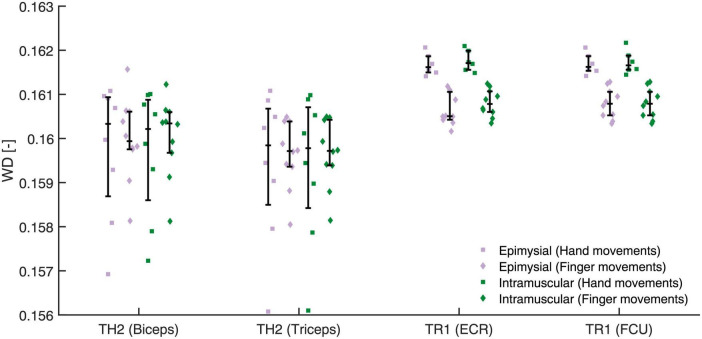
The Within-class Distance (WD) was calculated for either gross hand (square) or individual finger movements (diamond) using all electrodes except either the epimysial (violet) or intramuscular (green) electrode of the targeted muscle (shown in parenthesis next to the participant ID).

Repeatability calculated with WD for hand and finger movements is shown in Figure 6. For hand movements we found no difference between epimysial (0.161 [0.1597, 0.1615]) and intramuscular electrodes (0.161 [0.1597, 0.1616], *p* = 0.85). Similarly, for finger movements we found no difference between epimysial (0.1604 [0.1598, 0.1607]) and intramuscular electrodes (0.1605 [0.1599, 0.1609], *p* = 0.467).

The result of the sequential feed-forward selection can be seen in Table 2. No clear superiority of any electrode type is seen, but electrodes placed in the same muscle differ in ranking depending on their type. For TH2 the *Long Head Biceps* (LHB) epimysial electrode is the highest (hand movements) and third most contributing (finger movements), whereas the LHB intramuscular electrode is the third least contributing electrode. The same pattern is seen for *Long Head Triceps* (LHT) for the finger movements for TH2. For TR1 the intramuscular electrodes generally contribute more than the epimysial electrodes. However, the same pattern that was seen for TH2 is seen for TR1 for the finger movements where the intramuscular epimysial *Extensor Carpi Radialis longus* (ECR) electrode is the third most contributing electrode, while the epimysial ECR is only the eighth most contributing electrode.

**TABLE 2:** Results of sequential feed-forward selection.

Participant	Movements	← Highest contribution						Lowest contribution →					
TH2	Hand	LHB	–	–	–	LHT	–	–	–	–	LHB	–	LHT
	Finger	–	LHT	LHB	–	–	–	–	–	–	LHB	LHT	–
TR1	Hand	–	–	ECR	–	ECR	–	–	–	FCU	FCU	–	–
	Finger	–	–	ECR	–	–	FCU	–	ECR	FCU	–	–	–

Electrodes are ranked from left to right according to their contribution to the IDNN. Epimysial electrodes are marked violet and intramuscular electrodes are marked green. LHB, Long Head Biceps; LHT, Long Head Triceps; ECR, Extensor Carpi Radialis longus; FCU, Flexor Carpi Ulnaris.

## 4 Discussion

In this study, we conducted retrospective analyses of data from implanted epimysial and intramuscular electrodes to determine if either type is better suited for the control of bionic hands. Our analysis of electrode impedance shows that although impedances are generally higher for intramuscular electrodes than for epimysial electrodes (Figure 2), the ratio between cross-channel and direct impedance is also higher for intramuscular electrodes (Figure 3), suggesting that they may be better at isolating signals from a particular source without as much crosstalk from other sources. However, SNR analyses appeared to show no significant differences between epimysial and intramuscular electrodes (Figure 4), which may suggest that factors other than electrode type (such as training or muscle configuration) play a larger role in determining signal quality.

Separability and repeatability analyses more directly investigated the impact of electrode selection on factors influencing prosthetic control. Our results show no differences between epimysial and intramuscular electrodes in terms of separability and repeatability. This again aligns with the findings for SNR analyses suggesting that electrode type may play a smaller role compared to user skill or recorded muscles. It is important to note that the relationship between separability/repeatability and control is complex (Frankzke, 2023; Franzke et al., 2021) and that there might be a difference between electrode types in terms of controllability. Previous studies have found strong correlations between signal separability and offline classification, but weaker correlations to real-time control performance (Franzke et al., 2021; Nilsson et al., 2017). Studies differ, however, in their findings on correlations between repeatability and real-time control performance, with some studies showing stronger correlations (Bunderson and Kuiken, 2012; Nilsson et al., 2017) and others showing no significant correlations (Franzke et al., 2021). Future research should conduct real-time control experiments to determine how electrode selection influences prosthesis controllability.

The sequential feed-forward selection analysis gave an indication that some muscles contribute more depending on the electrode they are implanted with. As an example, larger muscles, like LHB, seem to contribute more with epimysial electrodes, whereas smaller muscles, like ECR, contribute more with intramuscular electrodes. One possible explanation could be that larger muscles benefit from the larger dimensions of the epimysial electrode, whereas smaller muscles, which lie in close proximity to other muscles, benefit more from the selectivity of intramuscular electrodes. Our analysis is only based on two participants, so more data is needed before any definitive conclusions can be drawn.

The selection of implantable electrodes warrants deliberate consideration to provide patients with the greatest potential for upper limb prosthetic control. Overall, we found few substantial differences in signal quality and electrical properties between implantable epimysial electrodes and intramuscular. Instead, we believe that primarily geometric factors – electrode size, electrode polarity (monopolar or bipolar), and proximity between target muscles – play the most significant role in determining signal quality and separability. Larger muscles, such as *biceps* and *triceps brachii* used for transhumeral prosthesis control, may be served better with epimysial electrodes; a similar trend has been observed in sEMG electrodes, where larger electrode diameters yielded higher SNR (Kim et al., 2020). Smaller muscles in closer proximity, such as *flexor digitorum profundus* used for control of multifunction hands, may instead benefit from the increased impedance ratios of intramuscular electrodes. Bipolar configurations benefit both types of electrodes and are preferable if enough contacts are available.

Furthermore, even the best electrode setup cannot counteract poor muscle control on the part of the prosthesis user. Myoelectric prostheses – especially multifunction bionic hands – might require substantial practice to control skillfully. This practice can be guided by the prosthetist upon delivery of the prosthesis, and may be further enhanced by gamified practice (Garske et al., 2021; Kristoffersen et al., 2021; Kristoffersen et al., 2022) or internal motivational factors such as competition (Earley et al., 2022b). Although simple opening and closing of the prosthesis can be learned fairly easily and intuitively by some (Bouwsema et al., 2010), control of additional functions such as wrist movement or discrete grasping patterns can be more difficult, especially with poor longitudinal signal repeatability. Most participants had at least 2 years of practice controlling their prosthesis with their implanted electrodes, which have demonstrated stable impedances over time and required only infrequent control algorithm retraining (Ortiz-Catalan et al., 2020; Ortiz-Catalan et al., 2023; Zbinden et al., 2023). However, we suspect that the low SNR during individual finger movements for TR2 was due in part to limited practice of those movements compared to other participants.

The present study is, to our knowledge, the first of its kind to compare chronically implantable electrodes *in vivo* in patients with upper limb amputation. A previous study had compared signal properties in *flexor carpi ulnaris* in a single able-bodied participant (Thorgeirsdottir, 2019); this study calculated similar impedances and SNR to those observed in the present study, but also generally observed no significant differences between the epimysial and intramuscular electrode in terms of electrical signal quality or pattern recognition performance. These findings, though preliminary, appear to align with the findings in the present study.

Our retrospective analyses should be considered exploratory due to low subject numbers and lack of prospective assignment and placement of electrodes. Because electrodes were selected based on functional considerations, accounting for factors including prioritizing muscles most used in the most functional movements, muscle sizes, and independence, our analyses were based on a convenience sample of available data from six participants. It is important to note that, while preoperative planning motivated the electrodes that were selected for implantation, the final decisions of targeted muscles were made during surgery and may have differed from the preoperative plan. Only two of these participants had been implanted with both electrode types, thus most comparisons made in this study are unpaired. That said, conducting a controlled and adequately-powered pairwise study of implantable electrodes in humans is infeasible. Furthermore, there are many factors that could influence signal quality between electrodes, including amputation level, muscle strength, presence of neuromuscular constructs, and participant skill with myoelectric control and willingness to practice to improve this control. However, our results may provide preliminary evidence that electrode selection may play an important role in specific situations.

Our analyses only considered two types of implantable electrodes, both from the same manufacturer, and thus our results may not generalize to electrodes made with different dimensions or materials. Another class of electrode which are also not considered in this analysis are nerve interfacing electrodes, which prior studies have shown may also be used for intent recognition for bionic limbs (Ahkami et al., 2022). However, since direct neural interfacing may present access to greater informational throughput than myoelectric electrodes, this comparison is outside of the scope of this study.

## 5 Conclusion

Implantable electrodes circumvent many of the limitations of surface electrodes via targeted recording of specific muscles. In this retrospective study, we analyzed data from implanted epimysial and intramuscular electrodes, and compared their signal quality and feature space characteristics, which are important qualities for the control of prosthetic arms and hands. Our results showed a higher ratio of cross-channel impedances to direct impedances for intramuscular electrodes, but this did not translate to improved SNR, separability, or repeatability. Sequential feedforward selection analysis may suggest that epimysial electrodes contribute greater signal separability when recording from larger muscles used for gross hand movements, whereas intramuscular electrodes contribute greater signal separability when recording from smaller muscles used for grasp prehension and finger movements, but additional study is required to confirm these findings. Our results provide a preliminary understanding as to which electrodes should be used for which patients and may help to guide clinical practice for future implementation of cutting-edge bionic arms.

## Data Availability

The raw data supporting the conclusions of this article will be made available by the authors, without undue reservation.
